# Cellular Response to a Novel Fetal Acellular Collagen Matrix: Implications for Tissue Regeneration

**DOI:** 10.1155/2013/527957

**Published:** 2013-07-22

**Authors:** Robert C. Rennert, Michael Sorkin, Ravi K. Garg, Michael Januszyk, Geoffrey C. Gurtner

**Affiliations:** Hagey Laboratory for Pediatric Regenerative Medicine, Division of Plastic and Reconstructive Surgery, Department of Surgery, Stanford University School of Medicine, Stanford, CA 94305-5148, USA

## Abstract

*Introduction*. PriMatrix (TEI Biosciences Inc., Boston, MA, USA) is a novel acellular collagen matrix derived from fetal bovine dermis that is designed for use in partial- and full-thickness wounds. This study analyzes the cellular response to PriMatrix *in vivo*, as well as the ability of this matrix to facilitate normal tissue regeneration. *Methods*. Five by five mm squares of rehydrated PriMatrix were implanted in a subcutaneous fashion on the dorsum of wild-type mice. Implant site tissue was harvested for histology, immunohistochemistry (IHC), and flow cytometric analyses at multiple time points until day 28. *Results*. PriMatrix implants were found to go through a biological progression initiated by a transient infiltrate of inflammatory cells, followed by mesenchymal cell recruitment and vascular development. IHC analysis revealed that the majority of the implanted fetal dermal collagen fibers persisted through day 28 but underwent remodeling and cellular repopulation to form tissue with a density and morphology consistent with healthy dermis. *Conclusions*. PriMatrix implants undergo progressive *in vivo* remodeling, facilitating the regeneration of histologically normal tissue through a mild inflammatory and progenitor cell response. Regeneration of normal tissue is especially important in a wound environment, and these findings warrant further investigation of PriMatrix in this setting.

## 1. Introduction

Extracellular matrices (ECMs) are used for a variety of surgical applications. However, differences in source species, tissues, and manufacturing processes can alter their *in vivo *physiomechanical properties [1–3], highlighting the importance of product choice in presurgical planning. While ideal ECM behavior varies for different clinical indications, it seems clear that products used for wound healing applications should act as a scaffold for host cellular infiltration and undergo progressive remodeling to form functional tissue without eliciting a foreign body or immunogenic response.

PriMatrix is a novel acellular collagen matrix that is designed for use in partial- and full-thickness wounds. PriMatrix ECM is produced from fetal bovine dermis, a rich source of type III collagen associated with wound healing and developing tissues [1, 4, 5], and is not denatured or artificially cross-linked during the manufacturing process [1]. PriMatrix is pliable following rehydration, allowing natural alignment to the wound site, yet remains strong enough to be sutured in place. Additionally, PriMatrix is highly porous, supporting the seeding of host cells that are thought to remodel and eventually completely replace the matrix with host tissue [6]. In fact, SurgiMend (TEI Biosciences Inc., Boston, MA, USA), a fetal bovine derived ECM similar to PriMatrix but approved for hernia repair and reconstructive surgery applications, has previously demonstrated a desirable host integration and adaptation response for up to 15 months in a rat subcutaneous implant model [1]. Nonetheless, a similar data and molecular characterization of the *in vivo *behavior of PriMatrix is lacking.

Clinically, PriMatrix has shown efficacy for the treatment of nonhealing, complex wounds in comorbid patients [7] and was also shown to significantly accelerate the healing of chronic neuropathic ulcerations compared to standard wound therapy [8]. Additionally, in a mid-sized retrospective comparison (68 wounds total) of PriMatrix to the Bilayered Living Cell Therapy Apligraf (Organogenesis Inc., Canton MA, USA) for the treatment of refractory diabetic foot and venous stasis ulcers, patients treated with PriMatrix displayed accelerated healing compared to those treated with Apligraf alone, despite larger initial wound size [9].

Given these promising pre-clinical and clinical results, this study aims to characterize the underlying cellular response and remodeling process of PriMatrix in a controlled *in vivo* setting, as well as to determine the ability of this matrix to facilitate normal tissue regeneration. 

## 2. Materials and Methods

### 2.1. Animals

Eight-to-twelve-week-old C57BL/6J mice (Jackson Laboratories, Bar Harbor, ME, USA) were utilized in this study. Animals were housed in the Stanford University Veterinary Service Center in accordance with NIH and institution-approved animal care guidelines, and all procedures were approved by the Stanford Administrative Panel on Laboratory Animal Care (APLAC).

### 2.2. PriMatrix Implantation and Tissue Harvest

A simple subcutaneous implant model was utilized, whereby three 5 × 5 mm squares of rehydrated PriMatrix were implanted on the dorsum of all mice, with one sham implant site serving as a control. Briefly, the mice were anesthetized and hair was removed from the dorsum, before being cleansed with three alternating scrubs of Betadine and 70% alcohol. In each quadrant, a horizontal 6 mm incision was created, and a subcutaneous pocket was bluntly dissected caudally in the fascial plane underlying the panniculus carnosus. A rehydrated PriMatrix implant was then inserted in three of the four pockets, and all pockets were irrigated with sterile saline before being closed using 6–0 nylon sutures. 

At days 1, 3, 7, 14, and 28 after implantation, a representative cohort of mice (*n* = 3) was sacrificed, and implant site tissue was photographed and harvested for histological analyses and flow cytometry. Care was taken to excise the implant and surrounding tissues intact. Intact skin and the sham-operated site without PriMatrix implantation were collected from all animals as controls. 

### 2.3. Histological Analysis

Collected tissue was harvested and immediately embedded in OCT (Sakura Finetek USA, Inc., Torrance, CA, USA). 10 *μ*m thick frozen sections were fixed in acetone and stained with hematoxylin and eosin (H&E, Sigma-Aldrich, St. Louis, MO, USA), Masson's trichrome (Sigma-Aldrich), or direct red 80 (picrosirius red, Sigma-Aldrich). Sections were also immunostained using antibodies against CD34 (1 : 100, Abcam, Cambridge, MA, USA), CD31 (1 : 200, Abcam, Cambridge, MA, USA) and bovine type I collagen (1 : 500, Millipore, Billerica, MA, USA), with nuclei stained with DAPI. Microvessel counts were conducted on intraimplant high power fields (400X) following CD31 staining. 

### 2.4. Flow Cytometry

For quantification of inflammatory and progenitor cell infiltrate following PriMatrix implantation, a portion of all excised experimental and control tissue samples were minced and incubated in 0.5 mg/mL Liberase TL (Roche Applied Science, Indianapolis, IN, USA) for 1 hr at 37°C and strained prior to staining. 

Unless otherwise noted, all antibodies were purchased from Becton Dickinson (Franklin Lakes, NJ, USA). For analysis of the inflammatory response, a portion of all samples were incubated with antibodies against CD45 (APC), CD11b (PE-Cy7), and F4/80 (eFlour 450, eBioscience, San Diego, CA, USA). The remaining samples were used for analysis of the progenitor cell response and incubated with a lineage negative (lin−) antibody cocktail (Ter119/CD4/CD8a/Gr-1/CD45R/CD11b, PE-Cy5, eBioscience, San Diego, CA, USA), as well as antibodies against CD45 (PE) and Sca-1 (FITC). 

Samples were incubated with antibodies for 30 minutes, washed, and resuspended in 2% fetal bovine serum in phosphate-buffered saline. Samples were recorded using an LSR Flow Cytometer (Becton Dickinson, Franklin Lakes, NJ, USA) and subsequently analyzed using FlowJo digital software (Tree Star, Inc., Ashland, OR, USA). 

For the inflammatory cell analysis, samples were gated for CD45+/CD11b+/F4/80− to define monocytes, and CD45+/CD11b+/F4/80+ to define macrophages. For the progenitor cell analysis, samples were gated for lin−/ CD45−/Sca-1+ to define mesenchymal progenitor cells.

### 2.5. Statistical Analysis

All values are expressed as mean ± SEM. Intact skin samples across time points were combined for analysis. Statistical significance across groups was determined using a one-way ANOVA, with subsequent comparisons between PriMatrix and control samples completed using a Tukey's post hoc analysis. *P* values ≤0.05 were considered statistically significant.

## 3. Results

### 3.1. Characterization of PriMatrix Biocompatibility and Remodeling

**Figure 1:**
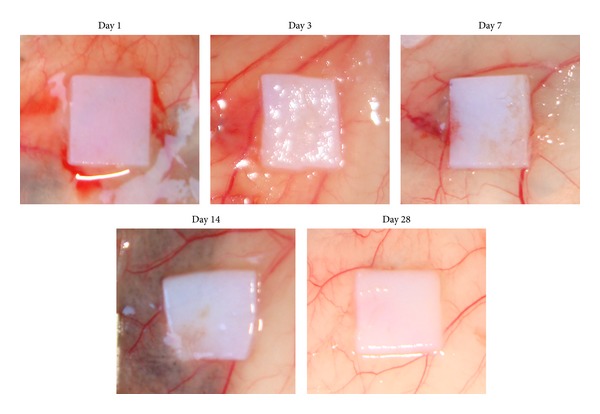
Gross photographs of PriMatrix implants at days 1, 3, 7, 14, and 28 following implantation. No inflammation or signs of rejection are observed; however, signs of implant resorption are indicative of a remodeling process.

**Figure 2:**
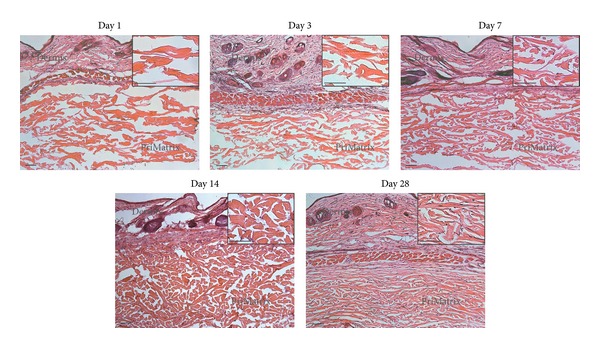
H&E staining of PriMatrix implants and overlying dermis at days 1, 3, 7, 14, and 28 following implantation (with insets of higher power images of implant). A progressive cellular infiltrate is seen populating the implant from the periphery, with the implant being remodeled to resemble the overlying dermis by day 28. At this time point, the transition from implant to native tissue is indistinct, with minimal fibrotic capsule formation noted. Scale bar is 100 *μ*m for all images.

To assess PriMatrix biocompatibility, we evaluated implants for signs of rejection, incorporation, and absorption *in vivo *using well-established parameters in the literature [1–3]. Upon gross dissection, no inflammation or signs of rejection were observed, and PriMatrix implants remained identifiable at day 28 (Figure 1). However, the implant displayed signs of resorption around the edges, consistent with a remodeling process. H&E staining was used to better characterize this response and identified the presence of a progressive cellular infiltrate into the implant from the periphery, which evolved to form tissue that was histologically indistinguishable from the overlying dermis by day 28 (Figure 2). Importantly, minimal fibrotic capsule formation was observed surrounding the implant at any timepoint, which overall displayed scant amounts of neovascularization and inflammation. 

**Figure 3:**
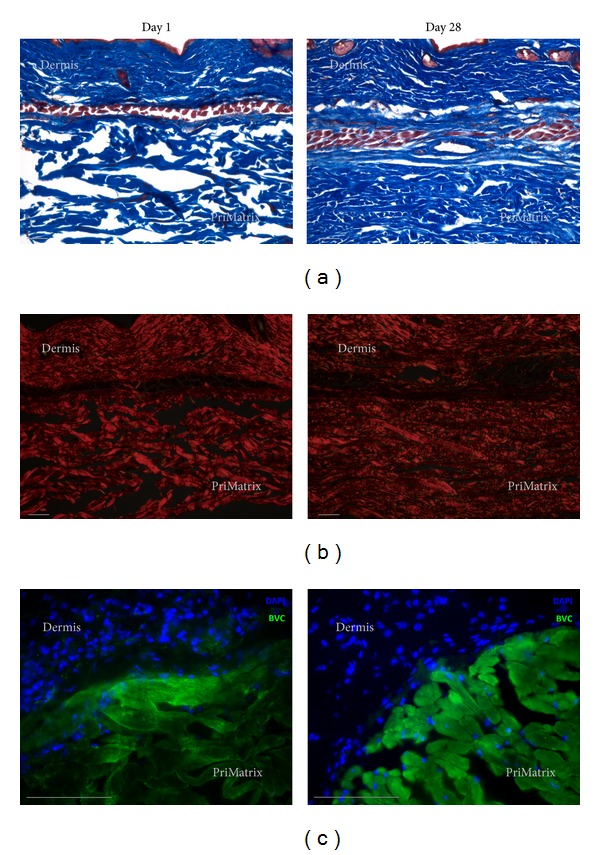
Characterization of collagen remodeling. Representative day 1 and day 28 images of PriMatrix implant and overlying dermis following trichrome (a) and picrosirius red staining (b), illustrating an increased density and organization of implants over time. (c) Representative high power images of immunofluorescence bovine collagen staining at days 1 and 28, demonstrating the persistence of PriMatrix derived fibers during the remodeling process. Scale bar is 100 *μ*m for all images. BVC: bovine collagen; DAPI: nuclear stain.

Trichrome and picrosirius red staining were then used to more accurately identify changes in implant collagen structure and demonstrated that the initially porous and disorganized implants were transformed to a density and organization resembling native dermis by day 28 (Figures 3(a) and 3(b)). To determine whether these changes were the result of PriMatrix remodeling or host collagen production, tissue sections were immunofluorescently stained for bovine collagen. Interestingly, while the PriMatrix architecture clearly changed over time, the observed persistence of bovine-specific collagen fibers (Figure 3(c)) indicated that the implant is remodeled during this process, but not entirely replaced with host collagen. 

**Figure 4:**
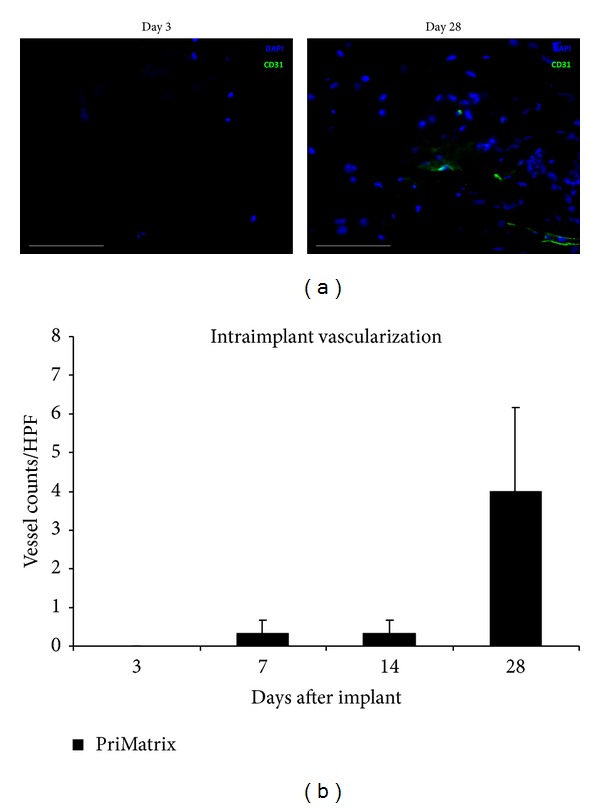
Characterization of intraimplant vascularization. (a) Representative high power images of intraimplant immunofluorescence staining for the endothelial cell-specific marker CD31 at days 3 and 28, showing foci of intraimplant neovascularization. (b) Quantification of intraimplant vessel counts per high power field. Scale bar is 100 *μ*m. DAPI: nuclear stain.

To evaluate intraimplant neovascularization, a crucial aspect of biomatrix *in vivo *adaptation, CD31 immunofluorescent staining, and quantification was conducted. Consistent with a functional remodeling process, a progressive increase in intra-implant neovascularization was observed, with the greatest vessel counts detected at day 28 (Figure 4). 

### 3.2. Inflammatory Response to PriMatrix Implant

**Figure 5:**
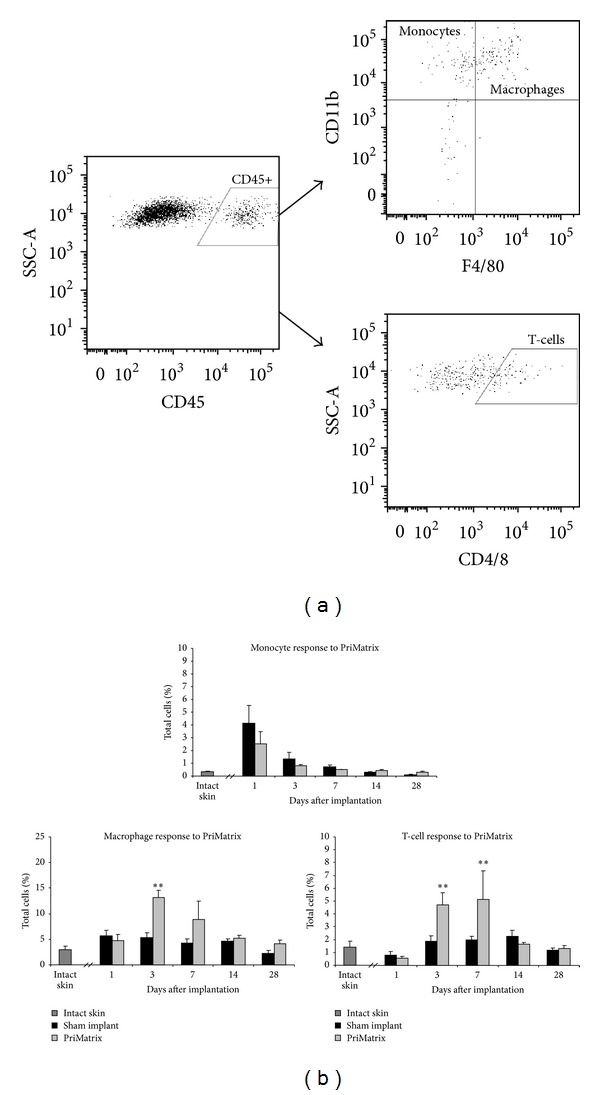
Flow cytometric analysis of the immune response to PriMatrix implants. (a) Gating scheme for monocyte, macrophage and T-cell analyses. (b) Quantification of the monocyte, macrophage and T-cell response, demonstrating an initial monocyte response on day 3, which transitions to a macrophage and T-cell dominated response at days 3 and 7. This inflammatory response was completely resolved by day 14, consistent with an adaptive tolerance to the implant. **Significant compared to intact skin and sham implant controls.

To further characterize the cellular infiltrate observed on initial H&E staining, flow cytometry for monocytes, macrophages, and T cells was carried out. This analysis revealed the presence of a mild, transient inflammatory response following PriMatrix implantation, which was characterized by an initial monocyte infiltration, followed by a macrophage and T-cell response (Figure 5). Consistent with our gross and histological observations of *in vivo *biocompatibility, this inflammatory response peaked on day 7 but was completely resolved by day 14. 

### 3.3. Progenitor Cell Response to PriMatrix Implant

**Figure 6:**
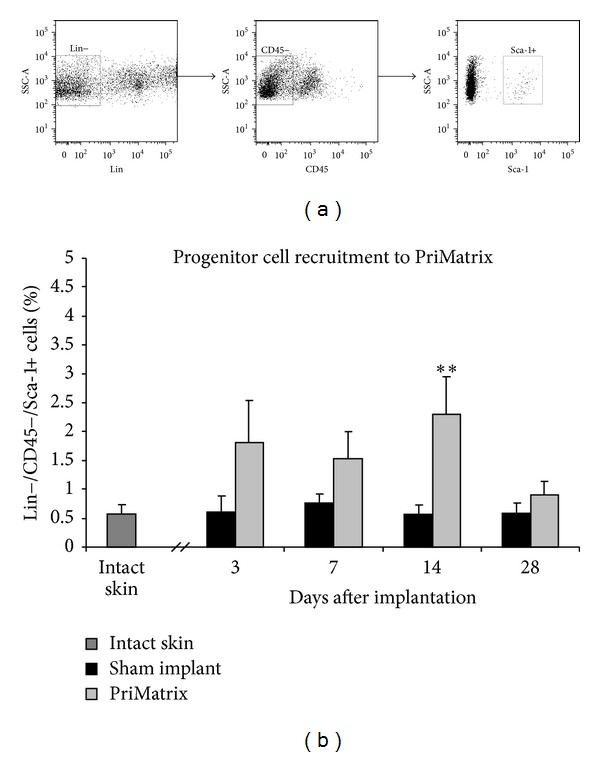
Flow cytometric analysis of the progenitor cell response to PriMatrix implants. (a) Gating scheme for the progenitor cell analysis. (b) Quantification of the progenitor cell response, demonstrating a significant increase in cell recruitment on day 14 compared to controls. **Significant compared to intact skin and sham implant controls. Lin: lineage staining.

Flow cytometry was also used to evaluate for progenitor cell recruitment into the PriMatrix implant. For this progenitor, cells were defined as being lin−/CD45−/Sca-1+. Consistent with a physiologic response, progenitor cell levels were significantly increased in PriMatrix samples on day 14 compared to intact skin and sham implant controls (*P* < 0.01) (Figure 6). Interestingly, progenitor cell numbers returned to baseline by day 28, suggesting a transient persistence of these cells in the implant.

To visualize the distribution of recruited progenitor cells within the implant and surrounding tissue, immunofluorescent staining for the general progenitor cell marker CD34 was then conducted. While the majority of CD34+ cells were located in the fascial plane directly adjacent to the implant, low levels of progenitor cell infiltration were also observed within the interior of the implant (data not shown).

## 4. Discussion

The host response and *in vivo* properties of ECMs are variable, reflecting the diversity in source tissue and processing techniques across products [1–3]. This range of biological performance likely makes some ECMs better suited than others for specific surgical applications, highlighting the need for an in-depth understanding of the biological response and *in vivo* characteristics of different products. 

The multitude of host responses to ECMs can be divided into two main categories: incorporating and nonincorporating [1]. Typical non-incorporating responses include encapsulation and rejection, which are both characterized by a moderate to strong inflammatory response and are often associated with artificially cross-linked products [1]. Cross-linking was initially done to mask foreign collagen antigenicity and prevent enzymatic digestion; however, these bonds can limit early host cell infiltration, ECM remodeling, and neovascularization, as well as increase the initial foreign body response leading to fibrous encapsulation [10]. While the host response to cross-linked materials may be advantageous in settings typified by the use of nondegrading polymers, such as abdominal wall hernia repair [11], it makes intuitive sense that wound healing and tissue regeneration applications require a product with more integration and remodeling capacities. 

ECM incorporating responses are likely better suited for these applications and include resorption, integration with progressive degradation, and adoption and adaptation [1]. These host reactions are more often seen with non-cross-linked ECMs and range from rapid resorption and replacement with scar tissue to integration of host cells and vasculature with either stable persistence or a continued slow degradation [1–3]. While incompletely understood, it is thought that many of the other differences across products, such as retained ECM components, sterilization type, and starting tissue source, are responsible for this variable host response [1, 3]. 

The use of ECMs for skin wounds is a relatively young field, with non-cross-linked human derived dermal matrices, such as AlloDerm (LifeCell Corp., Branchburg, NJ, USA) and, more recently, GRAFTJACKET (Wright Medical Technology, Inc., Arlington, TX, USA), showing efficacy for a variety of wound applications [12–15]. However, the optimal ECM in this setting remains unknown, as demonstrated by the variable *in vivo *responses and unclear clinical implications of recent comparative studies [2, 16].

PriMatrix has several potential advantages relating to its source tissue and manufacturing process, which may have contributed to the limited inflammatory response and capsule development observed in this study. Unlike adult derived products, PriMatrix is manufactured from fetal bovine dermis, with all lipids, fats, carbohydrates, and noncollagenous proteins removed during processing, leaving primarily type I and III collagen [1]. Additionally, PriMatrix collagen is not cross-linked during manufacturing, which likely contributed to the rapid intra-implant cell infiltration observed in this study and seen as early as day 7 on H&E staining. 

Not surprisingly, the *in vivo *behavior of PriMatrix is comparable to other TEI scaffolds derived from fetal bovine dermis. Specifically, the moderate inflammatory response to PriMatrix identified via flow cytometry was consistent with a previous histological characterization of TissueMend implants (a similarly derived matrix from TEI indicated for tendon repair), which demonstrated a transient inflammatory infiltrate and a long-term absence of foreign body giant cells [3]. In relation to the limited *in vivo *collagen remodeling displayed by the thicker constructs TissueMend and SurgiMend [1, 3], PriMatrix fibers similarly persisted following implantation but were remodeled to form tissue with similar collagen architecture as overlying dermis within 28 days. Finally, as previously demonstrated with TissueMend [3], histologic analysis of PriMatrix identified progressive, intra-implant neovascularization. This process is especially desirable for wound care applications, where ECMs can be even used in combination with an overlying split thickness skin graft [17], a technique that undoubtedly benefits from vascularization of the underlying matrix.

In contrast to the host inflammatory response and neovascularization potential of ECMs, the *in vivo *progenitor cell response to these products is less frequently studied. Extrapolating from the expanding evidence for endogenous progenitor cell recruitment following injury and involvement in neovascularization [18–20], we speculate that ECMs may provide a niche for progenitor cell engraftment, especially in the cytokine-rich wound environment. Supporting this hypothesis, the PriMatrix implants in this experiment demonstrated significantly increased levels of progenitor cell recruitment compared to both sham implant sites and intact tissue. While most of these cells were localized to the periphery of the implant upon IHC localization, intra-implant progenitor cells were also observed, and we suspect their numbers would be increased when subjected to the stronger cell recruiting signals present in a larger wound. Nevertheless, these findings must be tempered by the unclear implications of the transient nature of the progenitor cell response, which could be the result of cell efflux, death, or differentiation over time. The exact origin of these cells is also uncertain, as there is no universally accepted surface marker profile for different progenitor cell populations, and the definition used in the study did not distinguish between local cell migration and distal, blood-borne recruitment. Defining the lineage and functionality of recruited progenitor cells is therefore a crucial next step, for which more specific progenitor cell tracking models (such as parabiosis) [21] and surface marker definitions are required.

An additional caveat when extrapolating the results of this study is the use of a subcutaneous ECM implantation model, which may not predict PriMatrix behavior in the superficial wound environment. PriMatrix function was also not directly compared to other matrices, as this work was primarily intended to provide important initial *in vivo *biocompatibility and cell recruitment data. Nonetheless, these findings should help shape future studies attempting to determine the relative strengths and weaknesses of available ECMs. 

## 5. Conclusion

While a variety of dermis-derived ECMs are available for wound management and reconstructive applications, these products can generate divergent host responses based on differences in manufacturing and molecular composition. The ideal ECM for these applications would be immunologically tolerated, repopulated by host cells, and undergo remodeling similar to normal tissue. In this study, PriMatrix implants were found to undergo progressive *in vivo* remodeling, facilitating the regeneration of histologically normal-appearing tissue through a transient inflammatory and progenitor cell response. Future investigation of PriMatrix remodeling and host cell response in the wound healing setting is warranted based on these findings.
